# Age, gender, neck circumference, and Epworth sleepiness scale do not predict obstructive sleep apnea (OSA) in moderate to severe chronic obstructive pulmonary disease (COPD): The challenge to predict OSA in advanced COPD

**DOI:** 10.1371/journal.pone.0177289

**Published:** 2017-05-16

**Authors:** Xavier Soler, Shu-Yi Liao, Jose Maria Marin, Geraldo Lorenzi-Filho, Rachel Jen, Pamela DeYoung, Robert L. Owens, Andrew L. Ries, Atul Malhotra

**Affiliations:** 1 Department of Medicine, Pulmonary, Critical Care and Sleep Division, UC San Diego, San Diego, CA, United States of America; 2 Department of Medicine, UC Riverside, Riverside, CA, United States of America; 3 Pulmonary Division, University Hospital Miguel Servet, IISAragón, CIBERES, Zaragoza, Spain; 4 Pulmonary Division, Sleep Laboratory, Hospital das Clinicas, Faculda de de Medicina da Universidade de São Paulo, São Paulo, Brasil; Universidad Francisco de Vitoria, SPAIN

## Abstract

The combination of chronic obstructive pulmonary disease (COPD) and obstructive sleep apnea (OSA) is associated with substantial morbidity and mortality. We hypothesized that predictors of OSA among patients with COPD may be distinct from OSA in the general population. Therefore, we investigated associations between traditional OSA risk factors (e.g. age), and sleep questionnaires [e.g. Epworth Sleepiness Scale] in 44 patients with advanced COPD. As a second aim we proposed a pilot, simplified screening test for OSA in patients with COPD. In a prospective, observational study of patients enrolled in the UCSD Pulmonary Rehabilitation Program we collected baseline characteristics, cardiovascular events (e.g. atrial fibrillation), and sleep questionnaires [e.g. Pittsburgh Sleep Quality Index (PSQI)]. For the pilot questionnaire, a BMI ≥25 kg/m^2^ and the presence of cardiovascular disease were used to construct the pilot screening test. Male: 59%; OSA 66%. FEV1 (mean ± SD) = 41.0±18.2% pred., FEV1/FVC = 41.5±12.7%]. Male gender, older age, and large neck circumference were not associated with OSA. Also, Epworth Sleepiness Scale and the STOP-Bang questionnaire were not associated with OSA in univariate logistic regression. In contrast, BMI ≥25 kg/m^2^ (OR = 3.94, p = 0.04) and diagnosis of cardiovascular disease (OR = 5.06, p = 0.03) were significantly associated with OSA [area under curve (AUC) = 0.74]. The pilot COPD-OSA test (OR = 5.28, p = 0.05) and STOP-Bang questionnaire (OR = 5.13, p = 0.03) were both associated with OSA in Receiver Operating Characteristics (ROC) analysis. The COPD-OSA test had the best AUC (0.74), sensitivity (92%), and specificity (83%). A ten-fold cross-validation validated our results.

We found that traditional OSA predictors (e.g. gender, Epworth score) did not perform well in patients with more advanced COPD. Our pilot test may be an easy to implement instrument to screen for OSA. However, a larger validation study is necessary before further clinical implementation is warranted.

## Introduction

Chronic obstructive pulmonary disease (COPD) is a common, preventable and treatable disease that is characterized by persistent respiratory symptoms and largely irreversible airflow obstruction.[1, 2] COPD is a major global epidemic that occurs in >10% of adults over 40 years of age, accounts for >5% of physician office visits and 13% of all hospitalizations, and has become the 3^rd^ leading cause of death in the U.S.[1–6] Obstructive sleep apnea (OSA) is characterized by periods of partial or total intermittent collapse of the upper airway, resulting in nocturnal hypoxemia and arousals from sleep.[7–9]US data (2007–10) indicate an increasing trend with 26% of adults estimated to have mild to severe OSA, and even higher rates have been reported in some European Countries.[10, 11]

Flenley coined the term “overlap syndrome” when COPD and OSA coexist.[9] The prevalence of OSA among COPD subjects has been reported in different studies to range from 10% to 66%.[12–20] Studying the COPD-OSA syndrome is relevant as hypoxemia (both day and night) is more profound in patients with COPD-OSA compared with either condition alone.[15] Furthermore, mortality and morbidity are increased if OSA remains untreated.[21–23]

Given the impact of OSA in patients with COPD, it seems logical to investigate predictors that would help identify overlap subjects. Although age, gender, and neck circumference have been shown to be good predictors of OSA in epidemiologic studies, it is reasonable to hypothesize that these predictors may vary according to the specific population studied.[10, 24, 25] For instance, the impact of pathophysiological changes seen in more advanced COPD (e.g. chest hyperinflation) may impact mechanisms of OSA and potentially modify predictors of disease.

Several instruments have been developed to evaluate sleep quality and sleep disordered breathing (e.g. OSA). For example, sleep questionnaires have demonstrated clinical utility for OSA evaluation and are reviewed elsewhere.[26] Sleep quality can be assessed using the Pittsburgh Sleep Quality Index (PSQI) which provides a global index of sleep quality over the previous one-month interval.[27] The Epworth Sleepiness Scale (ESS) assesses daytime sleepiness.[28] Finally, a more recent instrument, the STOP-Bang, was developed to assess patients in the perioperative setting.[29] STOP-Bang is an eight-item survey combining symptoms (e.g. snoring) and clinical variables (e.g. BMI, age) and has been proposed as a potential screening tool in different clinical conditions such as obesity.[30]

First, given our previous findings of higher than expected prevalence of OSA among patients with moderate to severe COPD enrolled in a pulmonary rehabilitation program,[19] we sought to investigate predictors of OSA among this subgroup. We hypothesized that advanced COPD would not have the same predictors of OSA as in the general population mainly because of differences in: 1) upper airway dynamics; 2) respiratory pattern; 3) drugs involved in treatment; 4) OSA symptoms; and 5) associated co-morbidities.

Second, as a pilot study, we evaluated the overall performance of the ESS, PSQI, STOP-Bang and a new proposed pilot tool to screen for OSA in patients with moderate to severe COPD, using the presence of cardiovascular disease (e.g. atrial fibrillation), a common feature in OSA. As a result, we proposed a simplified two-question instrument that may help screen for OSA in patients with advanced COPD. We focused on this subgroup of COPD as those likely to benefit most from early diagnosis and treatment.

## Methods

This was a prospective, observational study of patients enrolled in the UCSD Pulmonary Rehabilitation Program during a two-year period from September 2010 to August 2012. Patients were screened and enrolled based on the following selection criteria: age ≥40 years and diagnosis of COPD by GOLD guidelines.[1, 2] The UCSD Human Subjects’ Protection Program approved the protocol and written informed consent was obtained from all subjects before the start of the study. We collected demographic and anthropometric measurements including, but not limited to: gender, age, body mass index (BMI), neck circumference, and the presence of prior cardiovascular events described as follows: being told by a doctor or having treatment for 1) systemic hypertension (HTN), 2) atrial fibrillation (AFib), 3) coronary artery disease (CAD), 4) peripheral artery disease (PAD), 5) or congestive heart failure (CHF). We assigned 1 point for a ‘Yes’ response to each of the 5 cardiovascular event questions. We also obtained ESS, PSQI, and STOP-Bang surveys from all study participants. All subjects underwent a full unattended home overnight PSG with a 16-channel portable system (Somte PSG, Compumedics, AU) to assess objective sleep-disordered breathing. All data were scored by a blinded certified registered polysomnography technologist (RPSGT) using the updated 2007 American Academy of Sleep Medicine (AASM) criteria.[31, 32] To identify all physiologically important respiratory events, the 2007 AASM alternative hypopnea definition was used (e.g. 50% reduction of airflow amplitude from baseline lasting ≥10 seconds associated with ≥3% oxyhemoglobin desaturation and/or an arousal terminating the respiratory event). Additional details about our scoring methodology can be found in previous publications.[19] OSA was diagnosed if the Apnea-Hypopnea Index (AHI) was ≥5 events per hour. Descriptive analysis was performed to compare OSA and No OSA groups. Univariate logistic regression analysis was used to evaluate the association between continuous variables and a diagnosis of OSA. Receiver Operating Characteristics (ROC) analysis was performed to evaluate the effectiveness of continuous variables including age, BMI, neck circumference, tobacco (pack/years), number of cardiovascular events, and sleep questionnaires (STOP-Bang, PSQI, and ESS) in detecting OSA. Odds ratios (OR) were obtained for each variable. Overweight was categorized by BMI ≥25 kg/m^2^; large neck circumference was defined as: male ≥17 inches (43.2cm) and female ≥ 16 inches (40.6cm).[33–35] Cardiovascular events were categorized as "Yes" with a positive answer to being diagnosed or treated for any of the cardiovascular conditions surveyed (HTN, AFib, CAD, CHF, PVD) and "No" if subjects denied all cardio-vascular conditions surveyed. Systemic hypertension was also analyzed as a categorical variable. Cut-points for the surveys were based on previous publications: ESS (cut-point ≥10), PSQI (cut-point >5), and STOP-BANG questionnaire (cut-point ≥3).[27–29]

Among all variables analyzed, BMI and the presence of previous cardiovascular disease were found to be significantly associated with OSA. Therefore, we developed a simple screening tool (COPD-OSA questionnaire) using a combination of these two variables. We assigned 1 point for a ‘Yes’ response to each question. The range for the pilot questionnaire was 0–2 with 1 point for high BMI and 1 point for the presence of any cardio-vascular disease. We performed a univariate logistic regression and maximum ROC analysis to evaluate the effectiveness of the proposed scoring tool. However, because the new instrument was based on the significant variables identified in one cohort, a further ten-fold cross-validation procedure in a different cohort was necessary to confirm our results. Cross validation is a testing method that allows one to confirm the results obtained from work with a relatively small number of subjects.[36] This procedure is a method to evaluate the predictive models by randomly dividing the samples into ten subsamples. A single subsample was reserved as the validation set for model testing, while the remaining nine subsamples were used as training set to train the model. This cross-validation procedure repeated ten times (folds), with each of the ten subsamples served as the training data every time. A single estimation was then obtained by averaging the ten results. We categorized the scores and analyzed results by univariate logistic regression models using different cut-points. The sensitivity and specificity of detecting OSA were calculated for each questionnaire. Statistical analyses were performed using SAS software (SAS Institute Inc., Cary, NC).

## Results

Fifty-four patients completed the baseline assessment. As expected, participants were generally older (age± SD = 67.2 ± 8.1 years), male (54%), and classified as moderate to severe COPD by spirometry;[1, 2] 37% were prescribed long-term supplemental oxygen at the time of the study. Three participants consented but dropped out during initial evaluation due to: COPD exacerbation (n = 2) or declined polysomnography (n = 1). Seven PSG studies (13%) were not suitable for analysis: sleep duration <4 hours (n = 2), loss of EEG signal (n = 2), and loss of raw data in a hardware malfunction (n = 3). A final sample of 44 subjects was included in the analyses. There were no significant demographic differences for patients who dropped out or who had inadequate PSG data. Baseline demographic, anthropometric, spirometric, and sleep questionnaires results are presented in Table 1.

**Table 1:** Characteristics of patients in the study.

Variable	Total	OSA	No OSA	P
	(n = 44)	(n = 29)	(n = 15)	
**Male (%)**	59.1	62.1	53.3	0.58
**Age (years)**	67.8±8.4	68.1±7.6	67.0±10.0	0.68
**BMI (Kg/m**^**2**^**)**	26.9±5.4	27.7±5.2	25.2±5.4	0.13
**Neck (cm)**	38.4±4.9	39.2±4.7	37.0±5.2	0.21
**Pack/year**	41.8±24.4	44.6±24.5	36.8±24.4	0.33
**FVC (% p)**	75.5±18.4	76.8±20.0	73.1±15.2	0.53
**FEV**_**1**_ **(% p)**	41.0±18.2	41.6±18.8	39.8±17.8	0.76
**FEV**_**1**_**/FVC (%)**	41.5±12.7	41.7±12.4	41.3±13.7	0.94
**STOP-Bang**	3.0±1.1	3.1±0.9	2.7±1.3	0.37
**PSQI**	8.3±4.3	7.7±3.9	9.3±4.9	0.26
**Epworth**	7.8±4.4	8.7±4.4	6.2±4.1	0.08

Definition of abbreviations: OSA = obstructive sleep apnea; P = P value; BMI = body mass index; Neck = Neck diameter. Pack/year = Pack/year of smoking; (% p) = Per cent predicted; Epworth = Epworth Sleepiness Scale PSQI = Pittsburgh Sleep Quality Index.

Traditional predictors such as male gender, older age, and large neck circumference were not associated with OSA in this cohort. In univariate logistic regression analysis of continuous variables, ESS, PSQI, and STOP-Bang questionnaires were not associated with OSA, with area under the curve of 0.64, 0.59, and 0.59, respectively (Table 2).

**Table 2:** Univariate regression and receiver operating characteristics analyses exploring the association of continuous variables and screening questionnaires (continuous scores) with obstructive sleep apnea.

Variable	OR	95% CI	P value	AUC
**Age**	1.02	0.94–1.10	0.67	0.51
**BMI**	1.11	0.98–1.29	0.24	0.65
**Neck diameter**	1.10	0.95–1.30	0.25	0.60
**Pack-years**	1.01	0.99–1.04	0.32	0.59
**Presence of CVD’s**	4.49	1.46–18.09	0.02	0.73
**STOP-Bang Questionnaire**	1.38	0.70–2.91	0.36	0.59
**PSQI**	0.92	0.78–1.06	0.26	0.59
**Epworth**	1.15	0.99–1.38	0.09	0.64

Definition of abbreviations: OR = odds ratio; CI = confidence interval; AUC = area under curve; BMI = body mass index; CVD’s = presence of cardiovascular diseases; PSQI = Pittsburgh Sleep Quality Index; Epworth = Epworth Sleepiness Scale.

Among all variables analyzed, BMI ≥25 kg/m^2^ and the presence of previous cardiovascular disease (HNT, AFib, CAD, CHF, PVD) were found to be significantly associated with OSA [BMI ≥25 kg/m^2^ (OR = 3.94, p = 0.04); cardiovascular disease (OR = 5.06, p = 0.03)]. Therefore, these two variables were used to construct a pilot-screening questionnaire. We assigned 1 point for a ‘Yes’ response to each question (BMI and presence of any cardiovascular disease). The range for the pilot questionnaire was 0–2 with 1 point for high BMI and 1 point for the presence of any cardio-vascular disease. The COPD-OSA questionnaire with cut-point ≥1 was associated with OSA (OR = 4.20, p = 0.04). With cut-point = 2, the questionnaire was also associated with OSA (OR = 5.28, p = 0.05), Table 3.

**Table 3:** Univariate regression exploring the association of categorical variables and screening questionnaire (with cut-points) with obstructive sleep apnea.

Variable	OR	95% CI	P
**Male**	1.43	0.40–5.13	0.58
**BMI≥25kg/m**^**2**^	3.94	1.09–15.51	0.04
Large neck circumference*	0.88	0.18–4.98	0.87
**Presence of CVD’s disease**	5.06	1.26–23.66	0.03
**Systemic Hypertension**	3.89	0.94–20.47	0.08
**STOP-Bang (cut-points ≥3)**	5.13	1.23–23.77	0.03
**PSQI (cut-points >5)**	1.43	0.35–5.68	0.61
**Epworth (cut-points >10)**	2.11	0.52–10.82	0.32
COPD-OSA (cut-points ≥1)**	4.20	1.06–18.11	0.04
COPD-OSA (cut-points = 2)**	5.28	1.18–37.80	0.05

Definition of abbreviations: OR = odds ratio; CI = confidence interval; BMI = body mass index. CVD’s = presence of cardiovascular diseases; STOP-Bang = STOP-Bang questionnaire; PSQI = Pittsburgh Sleep Quality Index; Epworth = Epworth Sleepiness Scale.

*Definition for large neck circumference: Male >17 inches (43.2 cm), female >16 inches (40.6 cm).

**COPD-OSA: COPD-OSA questionnaire is the pilot developed screening tool in our study with each point for cardiovascular disease and BMI ≥25kg/m^2^ (range 0–2).

The STOP-Bang questionnaire [cut-point ≥3 (OR = 5.13, p = 0.03)] was also associated with OSA. In the ROC analysis, the COPD-OSA questionnaire had the best area under the curve (0.74). Given the small sample for this pilot questionnaire, we performed a ten-fold cross-validation procedure to validate our findings (AUC = 0.65). The sensitivity and specificity of each questionnaire are summarized in Table 4.

**Table 4:** Accuracy of questionnaires as screening tools for obstructive sleep apnea including our proposed pilot COPD-OSA questionnaire.

Questionnaires	Sensitivity (%)	Specificity (%)
**STOP-Bang (cut-point ≥3)**	80	50
**PSQI (cut-point >5)**	72	25
**Epworth (cut-point >10)**	28	75
**COPD-OSA (cut-point ≥1)**	92	33
**COPD-OSA (cut-point = 2)**	52	83

STOP-Bang = STOP-Bang questionnaire; PSQI = Pittsburgh Sleep Quality Index; Epworth = Epworth Sleepiness Scale.

*COPD-OSA: COPD-OSA questionnaire is the pilot developed screening tool in our study with each point for cardiovascular disease and BMI ≥25kg/m^2^ (range 0–2).

The pilot COPD-OSA questionnaire with a cut-point ≥1 had the highest sensitivity (92%) in detecting OSA in patients with moderate to severe COPD, followed by the STOP-BANG questionnaire (80%) and PSQI (72%). The pilot COPD-OSA questionnaire with a cut–point = 2 had the highest specificity (83%), followed by ESS (75%). The number of cardiovascular diseases as a continuous variable (presence of 1, 2, 3, 4 or 5 surveyed diseases) for the COPD-OSA questionnaire improved the area under the curve (0.78) but had a lower sensitivity (56%).

## Discussion

Our study is important because it demonstrates that commonly used OSA predictors in the general population could differ in patients with advanced COPD. Risk factors such as being older, male gender, or large neck circumference were not associated with OSA in our cohort,[37–39] and, therefore, these predictors may not perform well and may not be clinically useful in COPD compared to non-COPD patients. Also, the Epworth Sleepiness Scale questionnaire was not associated with OSA. Perhaps patients with moderate to severe COPD are used to having poor sleep [40] and Epworth does not properly capture the degree of daytime sleepiness in these individuals because OSA symptoms present differently than in those without COPD. In contrast, both the pilot COPD-OSA test and STOP-BANG questionnaire were associated with OSA. However, the COPD-OSA screening test had the best area under the curve (0.74), sensitivity (92%) and specificity (83%).

Aging is a risk factor for OSA.[39, 41–44] Likely because our cohort had a high mean age (67.8 years) with relatively small standard deviation (8.4), we did not observe a major aging effect. OSA is also more common among men than women, although the gender differences are less marked after women go through menopause.[45] Women may be at greater risk of COPD than men for a given tobacco exposure, although the mechanisms are unclear.[46] In the present study, we did not find a major gender effect, perhaps because the majority of women were post-menopausal and the somewhat limited sample size. In theory, gender differences in apnea pathophysiology may be impacted both by aging and by COPD.[47]

Stradling, Davies, Katz and more recently Carmelli, found that a large neck circumference was predictive of OSA in the general population.[33–35, 48] In contrast, in our study of patients with COPD, neck circumference was not a good predictor of OSA. Neck circumference cut-points predicting OSA risk are ≥17” (43.2 cm) and 16” (40.6 cm) for males and females, respectively.[33–35] In our cohort, the mean circumferences were 40.7 and 35.1 cm for males and females, respectively, showing that OSA is common in patients with advanced COPD even among those with small neck circumference. The predisposition of COPD patients to OSA has been poorly studied to date, but may involve a complex interplay of anatomical and physiological factors.[49] Other factors (e.g., corticosteroids) that increase fat deposition in the neck leading to increased OSA risk may also contribute.[50] Mechanistically, the interaction between COPD and OSA may produce changes in airway collapsibility and ventilatory control compared to each disorder alone that overcomes neck size.[49]

As expected, we found that being obese was a risk factor for OSA (BMI ≥25 kg/m2). Bixler and coworkers found that BMI ≥31.1 kg/m^2^ in males and BMI ≥32.3 kg/m^2^ in females were associated with increased risk of OSA.[41, 42, 51] In theory, BMI in COPD may have a threshold effect such that patients with BMI ≥25 kg/m^2^ would have a higher risk of OSA. Also, it is plausible that the effects of air trapping on the upper airway could overcome (or compensate for) the effects of airway collapse due to obesity, [38, 44, 52] or emphysema.[20, 49] Further physiological studies may help explain such interactions.

We found that the performance of ESS, PSQI and STOP-Bang questionnaires in predicting OSA in advanced COPD was less than expected; STOP-Bang was found to be associated with OSA, but not the ESS or PSQI. The STOP-Bang questionnaire includes symptoms of snoring, observed stopped breathing, and tiredness. However, in our analyses, none of these symptoms was found to be associated with OSA. These findings are important because these tools are used commonly in clinical practice to assess OSA risk. Therefore, after finding that questionnaires such as Epworth did not predict the presence of OSA, we investigated, as a second and exploratory aim the use of an easy-to-use pilot screening tool for OSA using two components: 1) BMI (≥25 kg/m^2^), and 2) past medical history of any of 5 cardiovascular diseases: hypertension, atrial fibrillation, coronary artery disease, peripheral vascular disease, or congestive heart failure).

Tiredness, possibly a surrogate for fatigue, is a common symptom in patients with advanced COPD and may not be a good indicator of sleep disorders such as OSA.[40, 53] Because individuals with have poor sleep quality and disrupted sleep architecture, it is possible that such symptoms are in part a function of other common symptoms such as dyspnea or cough that disrupt sleep.[54–56] STOP-Bang includes the presence of systemic hypertension as a cardiovascular risk factor for OSA. Similarly, in our screening tool, we used the presence of any of five different cardiovascular conditions. However, and contrary to the STOP-Bang, we found that hypertension alone, a highly prevalent co-morbid condition, was not a predictor of OSA in moderate to severe COPD. About one third of patients with severe COPD may die from cardiovascular events, and, therefore, the presence of arrhythmias, stroke, myocardial infarction, or peripheral vascular disease may represent more accurately cardiovascular risks in this group of subjects. Another difference is that the BMI threshold for the STOP-BANG is >35 kg/m^2^; highly obese people are not commonly seen in advanced COPD and may explain some of the differences. Finally, the STOP-BANG questionnaire has an age lower limit (>50 years/old) and, although it may well represent the COPD population, it may miss younger persons with COPD (for which a cut-point is typically 40 years of age).

The ESS has been used for decades as a screening tool for OSA.[28] In the Sleep Heart Health Study, an increased ESS score was associated with severity of sleep-disordered breathing (SDB), habitual snoring, male sex, and other clinical symptoms.[57] In our study, specificity for ESS was 75% and we did not find an association with OSA. In fact, and consistent with the literature, Kapur and colleagues found in a community-based cohort that subjective sleepiness was absent in many individuals with SDB.[58] Although ESS grades the severity of sleepiness, this symptom alone may be very common in COPD and, thus, lacking in predictive value.[54]

Sleep quality was evaluated by the PSQI questionnaire where scores >5 are considered to reflect poor sleep. It measures subjective sleep quality with a symptom-based questionnaire that does not consider age, BMI, or history of cardiovascular diseases. In our study, we did not find an association of PSQI with OSA (Table 4). Sleep quality in COPD may be influenced by many factors. For instance, co-morbidities common in COPD, such as depression and anxiety, cause poor sleep quality and may be strong confounders.

There are several advantages to our proposed pilot-screening questionnaire. The simplicity and potential benefit would make it easy to implement in a clinical setting if larger validation studies confirm our findings. Furthermore, clinicians commonly collect such questions in regular encounters; therefore, little extra time would be required. Second, the screening test could be used to both rule in and rule out OSA. With a cut-point ≥1 as a positive test, we could exclude OSA in patients without associated or previous episodes of cardiovascular events and BMI <25 kg/m^2^ due to the high sensitivity. Also, we could rule in OSA with a cut-point = 2 if patients had both a past medical history of any the cardiovascular events described and a BMI ≥25 kg/m^2^.

We also acknowledge a number of limitations in this study. First, although we demonstrated that common predictors of OSA are not associated with OSA in these patients with advanced COPD, the relatively small sample size limits our ability to test of each type of cardiovascular event independently in our pilot-screening tool. Thus, we cannot be sure that each variable should have the same weight on the overall score.

Second, although to date this is the first proposed tool to screen for OSA in COPD, we only studied patients referred to pulmonary rehabilitation, which is known to be accessible to a minority of patients with COPD patients and may represent only a fraction of the COPD population. Although our patient population encompasses different degrees of disease severity, acculturation, and ethnicity, selection bias is also plausible. We deliberately studied the more severe spectrum of COPD, thus, our findings should not be generalizable to all COPD patients and, specifically, to those with mild disease. Studying patients with milder disease and from different centers will be required to validate and to expand upon these results.

Third, BMI is a dynamic measure that has some limitations. In fact, some individuals with COPD may have a low BMI and still have OSA.[19] Also, patients may have weight changes over time due to drug use (e.g. steroids), disease progression (e.g. muscle wasting), or tobacco cessation (reduced metabolism); therefore, evaluating dynamic OSA risk over time may be necessary. Our study population did not significantly changed weight after the pulmonary rehabilitation program (data not shown)

Fourth, in our study we used a low AHI cut-point of 5/h to define OSA because the presence of minimal respiratory events already triggers a sympathetic response with demonstrated systemic effects, in addition to some degree of hypoxemia found in many patients with advanced COPD. We believed that in this population it would be important to study sleep disorder disturbances with the minimal value of 5 as there may be important negative physiologic consequences in patients with mild OSA.[59] Although such detrimental effects in advanced COPD may potentially contribute to the increased morbidity and mortality seen in overlap COPD-OSA subjects, our work was not intended to answer this important research question. Future clinical trials will ultimately be required to determine the appropriate threshold for intervention. Based on our results, larger scale studies should be performed in a broader range of COPD subjects to validate and expand upon these results before recommending widespread implementation in clinical practice.

In conclusion, in this study we found that patients with moderate to severe COPD have OSA predictors that differ from those typically recognized in the general population. These findings are important in designing strategies to screen for important comorbid conditions found on these patients. In addition, we propose that specific tools are necessary to evaluate OSA risk in this population and we piloted a simple COPD sleep-screening questionnaire as a feasible instrument that may be applied clinically. However, since sleep disorders are very common among patients with COPD, further validation in a much larger cohort is necessary to confirm these findings and demonstrate its clinical utility. Furthermore, this area of research is underrepresented and additional efforts are warranted to help reduce the burden of disease from COPD.
